# Patient-centered practices for engaging transgender and gender diverse patients in clinical research studies

**DOI:** 10.1186/s12874-021-01328-4

**Published:** 2021-10-01

**Authors:** Andrew Asquith, Lauren Sava, Alexander B. Harris, Asa E. Radix, Dana J. Pardee, Sari L. Reisner

**Affiliations:** 1 The Fenway Institute, Fenway Health, 1340 Boylston Street, 8th Floor, Boston, MA 02215 USA; 2 Callen-Lorde Community Health Center, New York City, NY USA; 3 Division of Endocrinology, Brigham and Women’s Hospital, Diabetes & Hypertension, Boston, MA USA; 4 Department of Medicine, Harvard Medical School, Boston, MA USA; 5 Department of Epidemiology, Harvard T.H. Chan School of Public Health, Boston, MA USA

**Keywords:** Transgender, Clinical research, Public health

## Abstract

**Background:**

The purpose of this formative study was to assess barriers and facilitators to participation of transgender and gender diverse (TGD) patients in clinical research to solicit specific feedback on perceived acceptability and feasibility of research methods to inform creation of a multisite longitudinal cohort of primary care patients engaged in care at two community health centers.

**Method:**

Between September–November 2018, four focus groups (FGs) were convened at two community health centers in Boston, MA and New York, NY (*N* = 28 participants across all 4 groups; 11 in Boston and 17 in New York). FG guides asked about patient outreach, acceptability of study methods and measures, and ideas for study retention. FGs were facilitated by TGD study staff, lasted approximately 90 min in duration, were audio recorded, and then transcribed verbatim by a professional transcription service. Thematic analyses were conducted by two independent analysts applying a constant comparison method. Consistency and consensus were achieved across code creation and application aided by Dedoose software.

**Results:**

Participants were a mean age of 33.9 years (SD 12.3; Range 18–66). Participants varied in gender identity with 4 (14.3%) men, 3 (10.7%) women, 8 (28.6%) transgender men, 10 (35.7%) transgender women, and 3 (10.7%) nonbinary. Eight (26.6%) were Latinx, 5 (17.9%) Black, 3 (10.7%) Asian, 3 (10.7%) another race, and 5 (17.9%) multiracial. Motivators and facilitators to participation were: research creating community, research led by TGD staff, compensation, research integrated into healthcare, research applicable to TGD and non-TGD people, and research helping TGD communities. Barriers were: being research/healthcare averse, not identifying as TGD, overlooking questioning individuals, research coming from a ‘cisgender lens”, distrust of how the research will be used, research not being accessible to TGD people, and research being exploitative.

**Conclusion:**

Though similarities emerged between the perspectives of TGD people and research citing perspectives of other underserved populations, there are barriers and facilitators to research which are unique to TGD populations. It is important for TGD people to be involved as collaborators in all aspects of research that concerns them.

**Supplementary Information:**

The online version contains supplementary material available at 10.1186/s12874-021-01328-4.

## Background

Transgender and gender diverse (TGD) people are individuals whose gender identity differs from their assigned sex at birth. TGD people are disproportionately burdened by adverse outcomes across a range of physical and mental health conditions, such as in HIV infection, mental health, and substance use, and have unmet healthcare needs compared to their cisgender (non-transgender) peers [1–6]. Social marginalization and experiences of gender minority stress and stigma [7, 8] have been shown to fuel health disparities for TGD people. There is a need to identify health-promoting factors to leverage for interventions to improve TGD population health. Research has found that medical gender affirmation (hormonal therapies, surgical interventions) is associated with improved psychological functioning and health-related quality of life for TGD people [9–16], yet to our knowledge no studies have demonstrated a causal relationship between gender affirming medical care and improved HIV-related outcomes [17]. Studies are needed to evaluate the impact of medical gender affirmation on HIV prevention and care, known areas of health disparity for TGD people, particularly longitudinal research to assess improved outcomes over time to inform clinical care and models of healthcare delivery.

Despite the growing evidence-base that TGD individuals suffer profound health disparities, transgender individuals may be reticent to participate in research. TGD people face many barriers to access and receipt of healthcare generally, such as lack of knowledgeable providers, experiences of violence and harassment in health settings, and financial costs [4, 18, 19]. TGD individuals may experience apprehensions and/or similar barriers regarding participating in medical research about their health, such as lacking trust in cisgender researchers and providers due to concerns about damaging, exploitative encounters [20, 21]. Conversely, factors may facilitate the participation of TGD in research, such as access to needed medical care, community engagement and participation, and wanting to contribute to trans health research [21, 22].

Historically, TGD people have engaged with research in the context of HIV programs and funding resources. Some prior research has been conducted to assess barriers and facilitators to TGD people participating in HIV-specific research [21, 23, 24]. Aside from HIV clinical trials, there has been little insight into the barriers and facilitators of other health outcomes research in TGD populations [25, 26].

Research into barriers and facilitators to participating in research has been conducted in other marginalized populations, such as people living with HIV [27–29], men who have sex with men (MSM) [30–35] racial and ethnic minorities [32, 36, 37] and people who use drugs [32, 37]. Common barriers to participating in research for these groups have included: 1. Mistrust of researchers [38–41], 2. Feeling exploited/ having fears of being exploited [28, 41], 3. Aversion to research [42], 4. Time [32, 37, 40, 41, 43], 5. Study design concerns [44], 6. Not wanting to feel like a guinea pig [29, 37, 38, 40, 44], and 7. Confidentiality concerns [40]. Facilitators to research participation have included: 1. Finding meaning in the study content [38], 2. Believing the research could benefit participants and/or society/ altruism [31, 32, 37, 39, 42, 43, 45], 3. Financial incentive [31, 32, 34, 37, 39, 46], 4. Opportunity to build and/or be part of a community [32], 5. Familiarity with the organization conducting the research [31], 6. Having a trusted person, such as a primary care provider, think it is a good idea to participate [41], and 7. Having past positive experiences with the research staff [41].

One study examined barriers and facilitators to research participation in LGBT women living with HIV in Toronto, Canada, but it was not focused specifically on the trans community. This study found that “meaningful engagement” in research, especially for marginalized communities, required listening to the voices of the research participants. Participants spoke of facilitators to participating, such as feeling the research would come back to the community or benefit them, as well as barriers, such as feeling exploited or dehumanized when participating in research [28]. It is important to ascertain barriers and facilitators that exist for TGD people in research participation, especially any barriers and facilitators that uniquely necessitate consideration for the TGD population.

Additionally, the importance of study methods and procedures beyond surveys, such as collection of biospecimens for clinical research has been documented in prior research [47]. Certain health outcomes, such as STIs and viral load, cannot always be determined by self-report, and TGD specific biobanking efforts may provide scientific breakthroughs in understanding the relationships between medical gender affirmation and health outcomes [47].

The purpose of this study was to generally assess barriers and facilitators to participation of TGD patients in clinical research, and to solicit specific feedback on perceived acceptability and feasibility of research methods to inform creation of a multisite longitudinal cohort of primary care patients engaged in care at two community health centers. Prior research conducted by TGD researchers has advocated for this community input in the research process [48–50].

## Methods

### Study design

This qualitative study was designed to inform the research methods and content, study protocol, and infrastructure of a patient-centered longitudinal cohort study of TGD adult primary care patients at two community health centers. Between September–November 2018, four focus groups (FGs) were convened, two at Fenway Health in Boston, MA and two at Callen-Lorde Community Health Center in New York, NY (*N* = 28 participants across all 4 groups). The purpose of the FGs was to solicit community feedback, opinions, and suggestions about barriers and facilitators to research participation in TGD patients, and assess the perceived acceptability and feasibility of different research methodologies. Fenway Health and Callen-Lorde are community health centers with expertise in providing safe, competent, and informed care to LGBTQ populations [51]. In the last decade, the centers have served increasing numbers of TGD primary care patients. Across both sites, approximately 10,000 TGD patients received care in 2018.

### Participants and procedures

FGs were recruited using in-clinic print flyers, electronic advertisements on social media, and word of mouth between TGD patients and their providers; external recruitment was not necessary, as only current health center patients were eligible. Individuals who met the following criteria were considered eligible for participation: (a) age 18 years or older, (b) have a gender identity differing from their sex assigned at birth (verified at screening via two-step method cross-categorizing natal sex and gender identity), (c) current primary care patient at Fenway Health or Callen-Lorde (defined as those who have had at least one medical visit in the prior 12 months), and (d) able to read, speak and understand English.

Participants provided verbal consent on the phone prior to attending the FG discussion. They also completed a brief demographic survey (age, gender identity, sex assigned at birth, race, ethnicity, and geographic location) prior to FG participation. FG discussions were held in-person, lasted an average of 90 min and were led by a primary facilitator and a supporting facilitator Across each site, four TGD- research staff trained in focus group facilitation and qualitative interviewing methods facilitated the groups (self-identities of facilitators: 2 white TGD men, 1 white trans masculine genderqueer person, and 1 Black Afro-Caribbean woman of trans experience). Participants were compensated with a $25 gift card upon completion of each FG. The Fenway Institute Review Board approved all study procedures.

### Data collection instruments

A semi-structured focus group discussion guide was used to gather data across several domains: (1) perspectives on TGD research, (2) study participation, (3) study materials and communications, (4) recruitment, and (5) retention. FG guides were developed by TGD study team staff in collaboration with a multi-disciplinary team of investigators and a Community Advisory Board (CAB). FGs were audio recorded and transcribed verbatim by a professional transcription service.

### Data analysis

FG transcripts were analyzed using thematic analysis and applying the constant comparative method [52]. Using Dedoose 8.3.17 software, two independent analysts (self-identities of facilitators: 1 white cis woman, 1 white trans masculine genderqueer person, both experienced in qualitative research) applied thematic codes to a subset of transcripts and constructed a codebook informed by the focus group guide. Through an iterative process, analysts independently coded each transcript, and then compared codes for codebook refinement, integrating newly emerging themes. After each of the four FG transcripts were initially coded and reviewed, finalized codes were shared, and refined further to ensure consistency and consensus. All four focus group transcripts were re-coded with the finalized codes. The two analysts shared an initial list of themes with three other members of the research team who corroborated the themes (self-identities: 3 white TGD men, all experienced in qualitative work). Findings were also shared with the CAB and corroborated and cross-checked with study investigators and TGD community members. Sample demographics from the brief survey were summarized (frequency, percent) using Microsoft Excel.

## Results:

### Sample characteristics

Twenty-eight individuals participated in all 4 focus groups with 11 (39.3%) participants in Boston and 17 (60.7%) in New York City (Table 1). Participants were a mean age of 33.9 years (SD 12.3; Range 18–66). Participants varied in gender identity with 4 (14.3%) men, 3 (10.7%) women, 8 (28.6%) transgender men, 10 (35.7%) transgender women, and 3 (10.7%) nonbinary. Twelve (42.9%) were assigned male at birth and 16 (57.1%) were assigned female at birth. Eight (26.6%) participants were Latinx and/or Hispanic and 20 (71.4%) were not Latinx or Hispanic. Twelve (42.9%) participants were White, 5 (17.9%) Black, 3 (10.7%) Asian, 3 (10.7%) another race, and 5 (17.9%) multiracial.

**Table 1:** Descriptive Characteristics of Transgender and Gender Diverse (TGD) Study Participants (*N* = 28)

Characteristic	% (n) or M (SD), Range
Age in Years	33.9 (12.3), 18–66
Gender Identity	
Man	14.3 (4)
Woman	10.7 (3)
Transgender Man	28.6 (8)
Transgender Woman	35.7 (10)
Nonbinary	10.7 (3)
Sex Assigned at Birth	
Male Assigned at Birth	42.9 (12)
Female Assigned at Birth	57.1 (16)
Ethnicity	
Latinx/Hispanic	28.6 (8)
Not Latinx/Hispanic	71.4 (20)
Race	
White	42.9 (12)
Black	17.9 (5)
Asian	10.7 (3)
Another Race	10.7 (3)
Multiracial	17.9 (5)
Geographic Location	
Boston	39.3 (11)
New York City	60.7 (17)

### Motivators/facilitators to participating in TGD health research

**Fig. 1:**
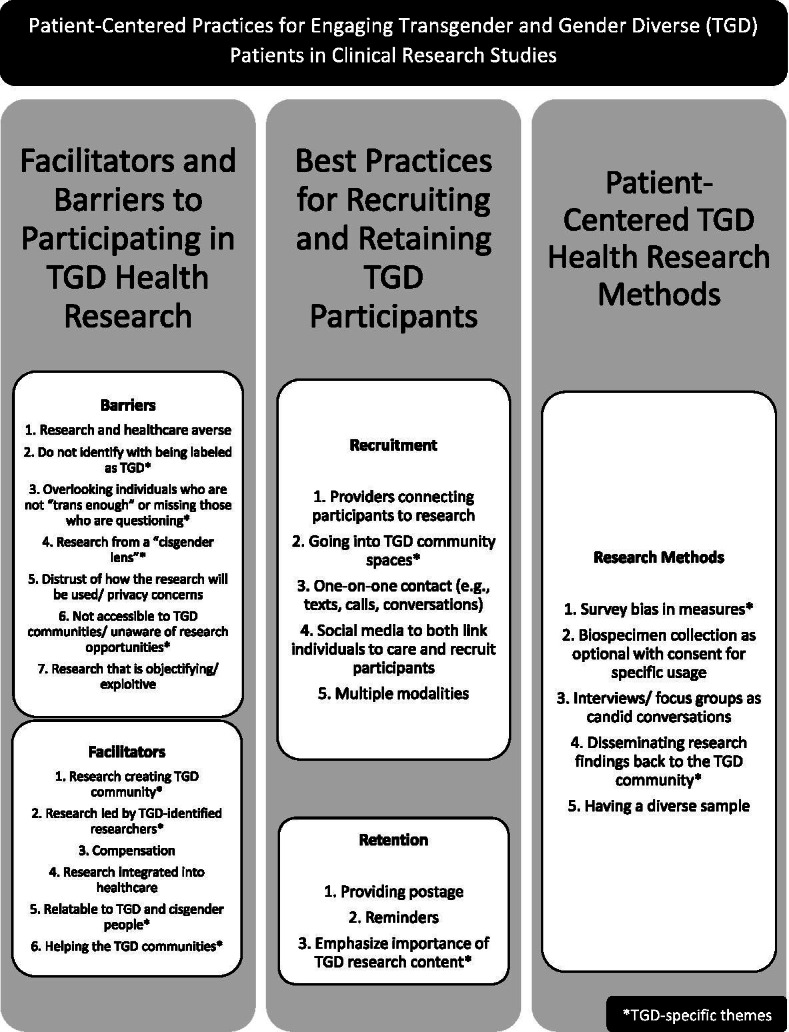
Patient-Centered Practices for Engaging Transgender and Gender Diverse (TGD) Patients in Clinical Research Studies

Participants discussed a variety of motivators/facilitators to participating in TGD health research projects (Fig. 1).

#### Research creating TGD community

Participants described that TGD health research may provide them the opportunity to connect with other TGD individuals, thus creating a community of participants. They described feeling motivated to participate in research as a means to feel engaged with other TGD community members (Table 2 Quote 1).

**Table 2:** Motivators/Facilitators and Barriers to Participating in Transgender and Gender Diverse (TGD) Health Research (*N* = 28)

**Motivators/Facilitators**	Research creating TGD community	1. “I know there’s a lot of situations where a trans person can become like – can just be isolated, but I think research and things that can help form a sense of community and, like you said, there have been so many millions of us throughout the years in human history.” -Boston
	Research led by TGD researchers	2. “I’ve done a few research things, and something I always appreciate is when they’re run by trans people. I had one thing where I didn’t know too much about the person that was interviewing me, and I was just, like, why is it not queer, like, not gay, not trans dude asking me all these, like, really [meaningful] questions about my transition? But then at the end, he did [say] that he was trans, and that just made me feel, like, a lot better. Because it’s like, talking about your experience.” -New York 3. “In terms of research priorities of being in research studies I feel like it’s a priority to have trans people and not just one token trans person but trans people as integral part of the research team designing it from the beginning. Like really there at every step.” -New York
	Compensation	4. “I was going to say, the only way to truly – to get more people would probably be to give them an incentive and to give them a gift card or something, because I get surveys all the time.” -Boston
	Research integrated into healthcare	5. “I think it’s great that it could just be integrated into our regular visits with primary care. We don’t have to really do anything super extra that would take up large chunks of our time, involve extra visits.” -Boston 6. “If you’d integrated it…that’s more efficient, yeah.” -Boston
	Relatable to TGD and cisgender people	7. “even a cis person, if you ask them about – something about how they relate to their body, it might get them thinking…Because I’ve read a lot that even with women who would consider themselves cisgender, they experience a massive amount of body dysmorphia just because of like how women – or cis women, rather, specifically, are forced to grow up and socialize and be sexualized from a young age and it creates like a really warped body image. Like, it’s something that I feel like a lot of women might not notice until you ask them about how they present themselves. And like, of course this manifests in trans women too because they’re still exposed to the same socialization.” -Boston
	Helping the TGD community	8. “I feel good about furthering trans-health research and trans-health equity.”” -Boston 9. “I would basically like to be going to the research studies, because it basically helps us trans people basically over time” -New York 10. “Yeah. I think research can be a form of advocacy if done correctly, and I think that anyone and everyone who participates in this study is doing their community’s a favor because I think data can be very powerful” -Boston
**Barriers**	Research and healthcare averse	11. “I’ve never done any studies anywhere. I’ve been told – two years ago, I never really saw doctors or went to hospitals for anything, for any reason whatsoever. I’m the type that, to even get me to walk into an ER, the bone’s got to be sticking out.” -Boston
	Do not identify with being labeled at TGD	12. “It’s complicated because it’s like good and bad at the same time. You know what I mean? But overall it’s just like – like I said there is no category for me. I don’t identify as any of that. I do but I don’t” -New York 13. “I don’t like to label myself as trans. You know what I mean? Like I don’t even like that word…can’t relate me to the community because I’m not the community. I am but I’m not. I’m just me. Don’t really identify.” -New York
	Overlooking individuals who are not “trans enough” or missing those who are questioning	14. “To build on that, I think maybe questioning folks may also not feel like they’re included…under the study premises even if they would be.” -Boston 15. “I would even argue that it’s important for people to identify that way or are stealth or – that data is there too. So it’s hard when you don’t want to share that and don’t identify as that but also that’s many people under the umbrella who should also be represented in some way.” -New York
	Research from a “cis lens”	16. “I tend to be instinctively kind of weary of trans TGD-focused things that aren’t headed at least mostly by trans people because it always feels a little bit like, “alright, so what are cis people gaining from this. Like, what is your stake in this?” And also it being led by trans people also makes me feel like there’s more of a floor to get really into the weird, granular stuff of gender experience without having –like, without the people you’re serving having to like dumb stuff down basically. At least don’t have to explain what trans means. [laughter] That’s big.” -Boston 17. “I feel like sometimes things that are meant to be more targeted towards the trans demographic sometimes kind of still feel like they have cis-people gloves on, if that makes sense. Kind of like baby talking through, and it’s like, “no, we can just talk seriously about this.”” -Boston
	Distrust of how the research will be used/ Privacy concerns	18. “it’s unfortunate when you’re dealing with any group of people that has been burned in the past. They just sometimes don’t trust easily. So they’re like, “Well, I’m not giving you my information.”” -New York 19. “I feel like there might be a chunk of people who don’t want anything to do with that study. Just be like, “I don’t want my information being out in the world.”” -New York 20. “people put in all these informations, and at the same time, you almost feel like it never reaches, like, people, so they can see us in a different light… So I feel like that’s maybe one of the reasons why they don’t want to participate.” -New York
	Not accessible to the TGD community/ Unaware of research opportunities	21. “Well, again, I’m just saying, what are we supposed to be doing, like, going to transresearch.com every single day [laughter]” -New York 22. “This was happenstance. I saw this on Facebook…I don’t know that transgender people – like, where are we supposed to hear about studies that are being conducted about our community?” -New York
	Research that is objectifying/ exploitive	23. “it felt like they were doing it because it’s the new hot thing and just wanted to like oh like this paper will get accepted, this is an easy project because there’s nothing about trans people so we don’t have to work too hard. And it really felt bad.” -New York 24. ““why are the results of this study so depressing?” Or like, “that’s not what I said. Why is what I said so miscommunicated in this report?” And it feels very exploitative and it feels very like we’re put under a microscope, right, as a community.” -Boston

#### Research led by TGD researchers

Many participants discussed wanting to participate in research that was led and facilitated by TGD researchers. They described how they would feel more comfortable talking about sensitive topics, such as gender affirmation and transitioning, with researchers who were TGD themselves (Table 2 Quote 2). Participants wanted TGD individuals to be involved in all levels of the research enterprise, not just as CAB members – but as leaders and part of the team to plan, develop, and implement the study research questions and protocols, collect data, conduct and interpret analyses, and publish and disseminate findings (Table 2 Quote 3).

#### Compensation

Another motivator/facilitator that participants frequently spoke about was receiving compensation (Table 2 Quote 4). Participants described monetary compensation such as gift cards or cash for their time. They also spoke of payment to compensate for costs incurred to participate, such as missing work or transportation costs and time. Participants indicated that larger incentives were necessary when the risks to participate were higher, such as with blood draws or sample collection. Additionally, participants highlighted non-monetary compensation, such as referral to other studies and health, social, or community resources, as facilitating participation.

#### Research integrated into healthcare

Participants explained that when studies are integrated into their healthcare visit or regularly scheduled appointments, it makes participating in research easier and more feasible (Table 2 Quotes 5–6). Leveraging existing visits prevents participants from having to make a special trip to the study site, and may reduce the amount of time required from the study participant, making the study more “efficient” (Table 2 Quote 6). Likewise, this approach of combining care and research may reduce barriers to participation, such as transportation and missed work.

#### Relatable to TGD and cisgender people

Some participants spoke of the importance of research content being relatable to both TGD and cisgender individuals. One participant gave an example of body dysmorphia being applicable to both TGD individuals and cisgender women, since cisgender women are often sexualized and experience unhealthy ideas of body image (Table 2 Quote 7). Some participants explained that research that spans health concerns of TGD and cisgender people would make the content more relatable and might provide motivation for participation in like-minded research.

#### Helping TGD communities

Many participants spoke of being motivated to participate in TGD health research to help the TGD community (Table 2 Quotes 8–10). Participants explained that research can help TGD individuals in similar ways to advocacy work (Table 2 Quote 10). They spoke of wanting to be a part of research that will impact their community in a positive way and to be altruistic.

### Barriers to participating in TGD health research

Participants discussed several barriers to participation in TGD health research projects (Fig. 1).

#### Research and healthcare averse

Some participants spoke of dislike and distrust of seeking healthcare, going to the doctor, medical environments, or participating in health research. One participant explained that they only accessed healthcare in dire situations; thus, they had never participated in research before (Table 2 Quote 11).

#### Do not identify with being labeled as TGD

A few participants spoke of not liking to be referred to as or not calling themselves TGD, “trans,” or “transgender.” One participant felt the word did not describe them (Table 2 Quote 12). Another explained this was not a part of their identity and they did not feel connected to or part of TGD communities (Table 2 Quote 13). These participants also described that they did not feel drawn to research labeled “transgender research” or studies marketed to TGD individuals.

#### Overlooking individuals who are not “trans enough” or missing those who are questioning

Participants expressed concern of TGD health studies overlooking individuals who are questioning or not “out” as TGD due to studies not recognizing them as TGD or the participant thinking they are not “trans enough” to be eligible for a study recruiting TGD individuals (Table 2 Quotes 14–15). One participant explained that many different identities would fall under the TGD umbrella and could be missed (Table 2 Quote 15).

#### Research from a “cisgender lens”

Participants expressed dislike of TGD health research where they had to simplify, over explain, or “dumb stuff down” for cisgender researchers (Table 2 Quotes 16–17). They also expressed being suspicious of why cisgender researchers were conducting TGD health research (Table 2 Quote 16). They described feeling how cisgender researchers may infantilize TGD people and may assume that TGD people are not knowledgeable about TGD health topics (Table 2 Quote 17).

#### Distrust of how the research will be used/ privacy concerns

Some participants expressed being wary of how research data will be used and not trusting researchers to give them their information (Table 2 Quotes 18–19). One participant explained it was because TGD individuals have been “burned in the past” by researchers (Table 2 Quote 18). Another individual explained that TGD participants often never see how their efforts benefit the community (Table 2 Quote 20).

#### Not accessible to TGD communities/ unaware of research opportunities

Participants expressed not knowing about health research opportunities or where to go learn about TGD studies (Table 2 Quotes 21–22). Participants also perceived that many TGD communities were often unaware of research studies.

#### Research that is objectifying/ exploitive

Participants described disliking research that felt opportunistic, wherein they perceived researchers were only conducting TGD health research because it was “the new hot thing” or publishable (Table 2 Quote 23). They also spoke of participating in research where they felt their experiences were misrepresented in research findings and disliked feeling like a subject or “put under a microscope” (Table 2 Quote 24).

### Best practices for recruiting and retaining TGD participants

Participants described “best practices” for recruitment and retention to meaningfully engage TGD individuals in research studies. These factors are displayed in Fig. 1.

#### Recruitment

##### Providers connecting participants to research

Many participants spoke of wanting to be referred to studies by their trusted medical providers. They suggested having providers give out fliers on TGD health research studies to their TGD patients (Table 3 Quote 1) or having pop-ups in provider emails to have them remind patients of study opportunities that patients may qualify for (Table 3 Quote 2).

**Table 3:** Patient-Centered Approaches for Engaging Transgender and Gender Diverse (TGD) Participants (*N* = 28)

**Recruitment**	Providers connecting participants to research	1. “I think having providers mention it would be helpful… like, “Okay, well since you’re here and you’re one of my trans patients, you might be interested in taking part in this study. Here’s a flyer.” Just kind of do it like that.” -New York 2. “What if, in terms of getting the little pop-ups in our inboxes about reminders, the providers also get little pop-ups in their inbox to remind their trans patients [of studies].” -Boston
	Going into TGD community spaces	3. “Well, a lot of us go to a lot of organizations or groups. So if you go to different organizations that are trans-focused and go to trans groups you’re going to find trans people.” -New York 4. “But as far as getting people out I guess can also have like flyers, information at places where trans people gather. Or maybe have someone come in and describe the studies to us. And then we’ll… see the person representing…so your organization must be good” -New York
	One-on-one contact (e.g., texts, calls, conversations)	5. “text messages’cause a lot of people are, like, always on the phone, so they’ll see.” -New York 6. “I also like this idea like sitting in person and having a conversation like face to face asking us how we feel about it.” -New York
	Social media to both link individuals to care and recruit participants	7. “Because, like, if you felt that you couldn’t get enough people at Callen, some form of social media might be able to get you the non-Callen trans people of New York.” -New York
	Multiple modalities	8. “Having multiple form of communications. Not just Facebook or digital but also paper forms. Phone calls. Text messages.” -New York 9. “I think also like using like phone call…very important for patients who have disabilities. If you just put posters up then blind people will never know it exists. So making sure that everybody can access the information even if they’re blind or deaf or whatever the case may be.” -New York
**Retention**	Providing postage	10. “You know, I take it if you mailed me a form, and at my convenience…Great, and the post is payed, put it in the box, that’s fine.” -Boston 11. “postage, paid, returned envelope… anyone who was remotely interested in their particular subject matter would [put] it in the mail.” -Boston
	Reminders (e.g., calls, emails, letters)	12. “Like an e-mail blast once a month. Nothing too aggressive. Just maybe every other month or once a month.” -New York 13. “Because it’ll show us you actually care…a check-in phone call in between visits.” -New York
	Emphasize importance of TGD research content	14. “Compensation is great, but also just being a part of it is really great because it’s for the advancement of our lives and a legacy that we can leave behind in a way. And so, maybe just the emphasis on that at the second interval.” -Boston

##### Going into TGD community spaces

Participants also highlighted the importance and acceptability of having researchers come to organizations, groups, and community spaces frequented by TGD individuals to either tell them about studies or pass out fliers (Table 3 Quotes 3–4).

##### One-on-one contact (e.g., texts, calls, conversations)

Participants expressed liking one-on-one contact methods. They explained this could be via texts sent out with study opportunities or having a face-to-face conversation about the study with a research staff member (Table 3 Quotes 5–6).

##### Social media to both link individuals to care and recruit participants

A few participants suggested that social media (e.g., Facebook, Instagram) would be an effective way to enroll TGD people in studies who are not currently accessing healthcare at study sites. Reaching out about TGD research opportunities was also described as a potential means of linking these individuals to needed healthcare services (Table 3 Quote 7).

##### Multiple modalities

Participants highlighted the importance of using multiple recruitment methods to reach TGD patients, such as telephone calls, texts, fliers, and social media (Table 3 Quote 8), especially from an accessibility standpoint (Table 3 Quote 9).

#### Retention

##### Providing postage

Participants felt that providing paid postage to return surveys and other forms of study data would make it easier to participate (Table 3 Quote 10–11).

##### Reminders

Participants expressed liking routine check-ins and contact with study staff via phone and email as reminders to participate in the surveys and visits, as well as to demonstrate to them that researchers care about their participants (Table 3 Quotes 12–13).

##### Emphasize importance of TGD research content

Some participants felt that TGD research content, specifically emphasizing how research participation can help TGD communities and society, can help to keep participants engaged (Table 3 Quote 14).

### Patient-centered TGD health research methods

Participants expressed ideas for improving research methods in TGD health research (Fig. 1).

#### Survey bias in measures

Many participants perceived that surveys and other measures in TGD health research tend to focus on negative outcomes and experiences. They spoke of how asking only “negative” questions, such as about depression and risks, may paint an overly negative picture of how someone is feeling, especially if they are not also asked about happiness, positive outcomes, or lived experiences of resiliency (Table 4 Quotes 1–2). One participant discussed the over-emphasis on gender dysphoria as an example of bias in TGD research, and wanted to learn about gender euphoria – a positive counterpart describing the feeling of self-actualization and joy in finding comfort in one’s gender identity and expression.

**Table 4:** Best Practice for Transgender and Gender Diverse (TGD) Research Methods (*N* = 28)

**Research Methods**	Survey bias in measures (e.g., quality of life scales focused on negative spectrum)	1. “Everybody was kind of nudging at this a little bit earlier and even just now, but there is a -- there’s like a scale for depression…I fill out this thing when I come into my PCP… there’s no inverse of that, you know what I mean? There’s no elation. [laughter] And I -- when you say we’re going to measure quality of life, I want to know how happy people are, you know?” -Boston 2. “Also, the thing about that is like I always feel like every single time I have to go to a PCP, I have to -- when I hand the tablet or whatever back to the nurse, I have to head it off with like, “I know this looks like I’m severely more depressed than I am.”” -Boston
	Specimen collection as optional with consent for specific usage	3. “at the very least say, “if we’re going to do something on it, we will come back to you and ask for that specific consent,” to say, “this is specifically what we’re going to do,” instead of being like, “take my DNA,”” -Boston 4. “it’s plasma; we’re not going to miss it much. But I guess…probably like clarity of purpose, or like communicating ideas of what you intend to do with it would probably be a better call.” -Boston 5. “about the blood collection…if you had just had it separated in two different parts, so it’s like, people that give blood and then people that don’t give blood.” -Boston
	Interviews/focus groups as candid conversation	6. “It was a cisgender man who identified as gay, and then me, I identify as genderfluid, and it was just, like, a candid conversation. We had never met each other, and it was us talking about health care and how we thought that things should change.” -New York
	Disseminating research findings back to the TGD community	7. “Right, and you’re left wondering, “what were the results of that study?”” -Boston
	Having a diverse sample	8. “I think other kinds of diversity is important. Not just like oh, it’s all trans people. Making sure it’s not all the same trans person. That it really represents the diversity of who we are.” -New York 9. “Well, you’d want to be able to get to multiple neighborhoods…for it to be a study that’s got meaning, you can’t be drawing from one strata, whether that means income strata, or one skin color…you’ve got to try to hit all those different rainbow colors…The people who are least likely to be able to be a part of this study are the people who you probably should want the most, in my opinion.” -Boston

#### Biospecimen collection as optional with consent for specific usage

Some participants expressed concerns about biospecimen collection. They wanted to know what the biospecimens would be used for and felt that researchers should acquire consent for each specific use of the biospecimen (Table 4 Quote 3). Others wanted to be told the purpose of collecting a biospecimen and how the researchers were planning to use it (Table 4 Quote 4). Some desired biospecimen collection to be an optional component or research study procedure (Table 4 Quote 5).

#### Interviews/focus groups as candid conversations

Participants expressed liking when interviews or focus groups felt informal and like a genuine conversation (Table 4 Quote 6). They also described the importance of transparency and comfort in these methods of data capture.

#### Disseminating research findings back to the TGD community

Participants explained that they wanted to be told about the research findings of the studies they participated in (Table 4 Quote 7). This was described as a way to build trust with communities and show respect for research participation. There was also an interest in ongoing dissemination of study findings, such as through quarterly or annual newsletters.

#### Having a diverse sample

Participants discussed the importance of including a diverse sample of TGD participants in TGD health studies. They wanted the sample to be representative of how diverse TGD individuals are (Table 4 Quote 8), as well as be inclusive of individuals from different neighborhoods, of different races and ethnicities, and with diverse LGBTQ identities (Table 4 Quote 9). One participant also emphasized wanting researchers to seek out hard to reach TGD individuals who “are least likely to be able to” participate in order to include their experiences and voices in the research (Table 4 Quote 9).

## Discussion

In discussing both facilitators and barriers to TGD health research participation, a consistent theme that emerged from this study was a strong desire from FG participants to feel connected to and engaged in the research, and certainty that the work would have an impact on TGD communities. Since the impact of research is often dependent on study findings, there is no guarantee as to what benefit findings can and will have on TGD communities. Therefore, there may be a need to provide education to research participants and TGD communities on research processes. This transparency is important for study participants having clear expectations and equipping them with better agency to decide whether or not to participate. Additionally, it is vital that researchers include TGD communities in conversations of how to best utilize research findings.

Participants highlighted that having TGD investigators and research staff leading the research would foster participant engagement and community comfort. A “participatory population perspective” has been described by Reisner et al. [53] as vital to public health efforts with TGD populations. This approach entails working “with” not “on” communities in public health research, practice, and advocacy. It is a methodology grounded in the philosophical perspective that any TGD public health endeavor will only be a true success if there is meaningful input and partnership with TGD communities. Findings from the current study support the use of a participatory population perspective to conduct clinical research with TGD people, including partnering with paid staff, researchers, and community members in all aspects of the work—research methodology, recruitment and retention, data collection, analysis and interpretation, and dissemination and sharing of results—to inform and advocate for TGD health justice.

Trust was a critical theme that emerged across focus groups. In the context of social stigma, many people who are members of a marginalized group, such as TGD populations, may feel most trusting of and comfortable interacting with people from within the same group. A barrier identified to research participation was the historical absence of TGD people on research teams. Participants felt that involvement of TGD staff would help to ensure a study is properly vetted and prioritizes participant safety, comfort, privacy, and trust. Lack of TGD research staff contributed to participant skepticism about whether and how the research is valued by the researchers or how findings will be utilized. Rather than feel like collaborators, participants may feel like they are being taken advantage of or exploited [41, 45]. While FG participants expressed concern over their experiences being commodified by research scientists seeking to advance their own agenda and cisgender lens, TGD-centered research is in actuality grossly underfunded and under-published. Additional TGD research is greatly needed, especially from funding streams beyond those focusing exclusively on HIV outcomes. Addressing these barriers is essential for making research careers accessible to more TGD individuals, which study participants expressed was essential in their comfort to participating in research. In addressing concerns of feeling exploited, it also important to consider and utilize multiple research methodologies. Depending on the research question and current level of trust with the community, it is imperative that research methods are employed thoughtfully and intentionally, and that researchers consider having a collaborative discussion (e.g. pilot acceptability and feasibility study, CAB) with community members before immediately recruiting TGD individuals to participate in a clinical study.

Building upon this theme, participants expected to be fairly compensated for their time, or to otherwise have participation be low-barrier and low-effort [31, 32, 34, 37, 39, 46]. For a cohort that is recruited from a clinical patient population, participants identified one way to make participation low-effort is to integrate survey measures into existing healthcare models and delivery. One example would be to not require additional visits to the clinic, if not necessary; however, there was a strong preference for compensation among participants, regardless of low-effort or streamlined survey implementation.

In addition, participants emphasized a desire for research methods that are non-pathologizing of TGD identities. Specifically, participants requested questions that could be asked of both TGD and cisgender TGD people, so as not to pathologize learning about TGD health and medicine. Further, they highlighted that some TGD people do not identify with the label “TGD” and therefore wanted to feel as though the questions being asked of them could apply to all people, irrespective of TGD status. Additionally, results underscore how some TGD people may still be questioning, unsure of their gender identity, not “out’ as TGD, or not using the identifier “TGD” to describe their lived experience. As a result, they may not understand that a study is inclusive of them, or may not participate due to internal stigma, even if the inclusion criteria are broad enough to encompass their gender identity. Participants expressed concern that individuals who do not perceive their own experience as “trans enough” may not participate—which could lead to survey bias and exclude information from an important segment of TGD people receiving clinical care.

Several themes that arose in the focus group discussions have important implications for recruitment and retention methods. Many revolve around the value of connection and building trusted relationships and rapport into recruitment and retention strategies. Participants spoke of wanting to be connected to research by their medical providers, a trusted party. They wanted researchers to similarly make a connection with them, either through one-on-one methods, such as telephone calls and texts, going into and showing up in TGD community spaces, or using social media networking platforms to reach out to them. This finding is important as we know that mistrust of researchers is also a common barrier to research participation among other minority populations [39–41, 54]. Methods that help build rapport between the community and researchers, therefore, are critical for TGD health researchers. Similarly, participants wanted to feel a connection to the study, explaining that the research content would likely keep them retained and engaged in research. Feeling they were a part of something that would make an impact was described as necessary to enhance research participation, a finding that has been observed in other minority populations [54]. Therefore, recruitment strategies—such as flyers and other recruitment materials—may benefit from highlighting the expected impact of a study for TGD people. In order to reach patients of all TGD experiences and identities, a multi-faceted approach is needed. Trust, skilled community engagement, inclusive eligibility criteria, inclusive recruitment language, and focused efforts to engage TGD subgroups are all methods of ensuring a strong sampling methodology.

Practicality emerged as another key theme for recruitment and retention of TGD people. Participants wanted researchers to reach out to potential study participants via multiple modalities. This was perceived as especially important to reach participants of different identities and create a diverse and inclusive sample. Multiple modalities of communication (e.g., flyers, email, text, telephone) were also mentioned as accessible ways of reaching TGD participants. Ease of participation was highlighted as important for recruitment as well as retention. Methods like providing postage and reminders via telephone calls, emails, and mail-out letters were identified as ways to make ongoing participation more realistic. Thus, TGD health researchers should prioritize methods to make participation in research as convenient as possible for participants, especially given many participants may have competing needs (e.g., work, family, housing). Ensuring ease of participation was also felt to be essential to building trust with participants. Methods that communicate and show participants that researchers value their time, a barrier to research participation documented in other marginalized communities [32, 37, 40, 41, 43], were highly endorsed. The themes of connection, rapport, and trust in TGD health research also emerged in discussing best practices for research methodology. Participants wanted qualitative research methods (e.g., focus groups, interviews) to feel candid, informal, and transparent. They also emphasized the import of disseminating research findings back into the community and prioritizing report-backs (e.g., newsletters with research results) as a means to show value and respect for research participation, which can be implemented most successfully when trust and rapport have already been established between researchers and participants. While many of these recruitment methods are applicable to other marginalized groups, it is still important for researchers working with TGD populations to utilize them, as they were confirmed by TGD focus group participants as priorities.

Participants were concerned that current research methodologies could be misrepresenting TGD people’s experiences and identities. They spoke of survey bias and measures often focusing exclusively on negative issues, such as depression, and missing the resilience of participants, such as learning and growing through hard times. The participant who described the focus on gender dysphoria at the expense of gender euphoria offered a powerful example. Findings highlight that research surveys which capture a full spectrum of participants’ experiences and feelings may maximize acceptability of research to TGD people. Further, researchers should consider adding resiliency scales to their surveys. Participants also expressed that researchers take care to ensure TGD research participants are not homogenous in identity, underscoring the importance of enrolling samples diverse in race, ethnicity, geographic locales, and sexual and gender identities.

Participants also emphasized building options into research methodology, such as biospecimen collection being optional with consent required for each specific usage. Participants wanted to be explicitly told how their biospecimen would be used. Research ethics guidelines dictated by the governing IRB do require informed consent for biospecimen [55]; however, participants expressed the desire for this consent to be more detailed and encompassing. They wanted to provide consent for each specific usage of their biospecimen and for researchers to ask their permission. In addition, participants wanted to have the option to opt out of biospecimen collection entirely while still having an opportunity to participate in other aspects of the study. Researchers should consider ways to offer participants more autonomy and control in decision-making about participating in research, including opting not to participate in some components. This further serves to communicate respect for TGD people in clinical research procedures and re-enforces trust-building with the community.

Amongst barriers and facilitators to participating in research, participants discussed financial incentive. It is important for participants to be compensated fairly for their time as they may need to take time off work and pay for transportation or childcare to participate. However, researchers also need to consider what is a reasonable amount so individuals with financial hardship do not feel obligated to participate.

Our findings with TGD patients corroborate findings from prior research on research participation in other marginalized populations. There are common facilitators (e.g., financial incentive/ compensation, altruism/ helping their community, positive experiences with research staff) and barriers (e.g., study design concerns, feeling exploited, confidentiality concerns) to research participation amongst other marginalized communities. However, this study also found that there are barriers and facilitators unique to TGD populations. The unique facilitators included research led by TGD researchers and being relatable to TGD and cisgender people. The unique barriers were not identifying with being labeled as TGD, overlooking individuals who are not “trans enough” or missing those who are questioning, and research from a “cisgender lens.” Unique research methods were also identified, such as survey bias in measures. Therefore, it is important for TGD voices to be involved in all aspects of research, including the planning, study design, recruitment, data collection, analysis and interpretation, and dissemination. It is important to identify research priorities for TGD people, including where these priorities do and do not overlap with other stigmatized groups. However, identifying best practices for research with TGD people ultimately requires data from TGD research, rather than applying research from non-TGD groups. Many of these themes are in alignment with research led by other TGD researchers advocating for community input in research, sharing research findings back to the community, having a diverse sample of TGD identities, using TGD affirming language, and empowering TGD individuals to lead and provide input on TGD research [48–50].

### Limitations

Interpretation of study findings should be contextualized alongside several limitations. First, the study had a small sample size. Saturation was attained with participants from the same sites sharing the same themes. Despite this, the findings may not resonate with transgender individuals who were missed during recruitment. While diverse in terms of age, gender identity, sex assigned at birth, and race, FG participants were not sampled in a representative fashion so may not reflect the patient populations of clinical sites. Additionally, the majority of focus group facilitators were white which may have been a barrier to participation or engagement of TGD people of color; non-white participants may not feel as comfortable sharing their experiences openly with white staff. This work is based in the US and framed accordingly. Participants were sampled from two major cities on the east coast and therefore, may not be generalizable to transgender individuals living in other regions of the US or less urban areas. We suggest future work expand to other regions in the US and internationally, as well as discuss global models of care, with attention to local and country context, culture, and medical systems. More research should also be conducted to understand how to engage and reach TGD people not currently engaged in medical care. Research is needed with hard to reach TGD individuals, purposively sampling those who are questioning and less connected to TGD communities. Due to focus groups being conducted in-person, accessibility issues may have limited participation; multiple methods, including online participation, should be considered for future research. Despite limitations, this study has a number of strengths, including assessment of barriers and facilitators to participating in clinical research for TGD people by asking TGD participants themselves.

## Conclusion

Although the list of barriers and facilitators identified to research participation for TGD people in this study is not exhaustive, it is a promising starting point for future researchers to consider when developing projects that engage TGD communities. More time should be spent to continue learning about and overcoming the barriers and facilitators TGD people face to participating in research, as well as how research methodologies, recruitment, and retention efforts can best engage and reach TGD individuals. Results suggest that gender-affirming practices grounded in community engagement and participation, transparency, and trust are vital to TGD research. Working collaboratively in researcher-community partnerships to move TGD health research ahead should be prioritized as a strategy moving forward in TGD clinical research.

## Supplementary Information

**Additional file 1.**

## Data Availability

The datasets used and/or analyzed during the current study are available from the corresponding author on reasonable request.

## Abbreviations

TGD: Transgender and gender diverse
FG: Focus group
MSM: Men who have sex with men
CAB: Community Advisory Board
