# Supplementary material for: Patient-centered practices for engaging transgender and gender diverse patients in clinical research studies
Source: BMC Med Res Methodol. 2021 Oct 1;21:202. doi: 10.1186/s12874-021-01328-4 (PMC8487157; doi:10.1186/s12874-021-01328-4)
Supplement: Supplementary file 1 — Additional file 1. [file 12874_2021_1328_MOESM1_ESM.docx]

Transgender cohort study of gender affirmation and HIV-related health

**Semi-structure Focus Group Guide**

**Version 1.0 | June 6, 2018**

Hello, my name is **[facilitator name]** and I will be facilitating today’s discussion. My co-facilitator is **[Name]**.

Thank you for taking the time to participate in this research! As a reminder, the goal of this focus group discussion is to get your feedback, opinions, and suggestions on the methods we will be using an upcoming study at Fenway Health in Boston, MA and Callen-Lorde Community Health Center in New York. This study will form a cohort of 4,500 transgender patients between the two sites that we will follow for one year to look at how gender-affirming care impacts health outcomes.

In our focus group discussion today, I will ask questions that relate to the upcoming research, such as questions about recruitment, marketing and study materials, and acceptability of study surveys and instruments. Your insights and viewpoints will inform the next phase of the project, which is the launching of the study cohort.

Please remember that you can refuse to answer any questions that make you feel uncomfortable, and you can leave the conversation at any time. There are no right or wrong answers to any of the questions involved in our discussion today, so please feel free to speak openly and honestly. All ideas are equally valid and this is a safe space to discuss ideas together. We want to learn about your experiences and hear your opinions.

As a reminder, I will be audio recording this session so I can focus on our discussion without having to take too many notes. Please try to speak one at a time so that we can hear everyone’s opinions. No one outside of this study will have access to these recordings and they will be de-identified. Please try not to use names or say other identifying information when you speak.

Do you have any questions before we begin?

**[TURN ON AUDIO RECORDER]**

OK, this is [**facilitator initials**] at **[Fenway Health or Callen-Lorde]** conducting focus group discussion **[focus group ID]** on **[date]** at about **[time].**

- ***The facilitator will begin by asking participants to introduce themselves by first name or nickname only and reminding all participants of the consent process, including requests to maintain other participants’ privacy.***
- ***Ground rules***

**NOTE TO FACILITATOR:** Primary topic area appear in **bold**. “Probes” appear with bullet points and provide some examples of topics that may require follow-up. Probes can help solicit additional detail/clarification or help move the conversation forward. Remember to pause after questions/probes to allow participants to think and respond with detail.

**1. OPENING DISCUSSION: Let’s begin by talking about what gender-affirming health care means to you.**

- *What comes to mind when you think of gender-affirming health care?*
- *What are some examples of times you have felt affirmed in your gender when seeking care?*

**2. RESEARCH PERSPECTIVES: I’d like to hear about your perspectives and experiences with research, including whether or not you have participated in research projects before. I’d also like to hear your perspectives about transgender people and research more generally.**

- *Have you, personally, ever participated in a research project? If yes, what was the experience like? What did you like? What did you not like?*
  - *If you have not personally participated in research, why not? What might make you want to participate in the future?*
- *Why do you think that transgender people do and/or do not participate in research?*
  - *What do you see as barriers for transgender people to participate in research?*
  - *How might these barriers be overcome?*
  - *What do you see as facilitators (things that make it easier) for transgender people to participate in research?*
  - *How might these facilitators be enhanced or built upon to make it easier for transgender people to participate in research?*
- *From your perspective, what are the top research priorities in transgender health?*

**Before we continue the questions, I would like to tell you a little bit about the study that we are planning to implement, so that you can give us your opinions and help to guide the research. Current and new transgender patients at Fenway Health and Callen-Lorde will be invited to the study. We are aiming to enroll 4,500 patients between the two sites. All participants will undergo an informed consent process, and complete a brief baseline survey. We will follow people for a period of 12 months. Participants will be asked to fill out a brief survey at 6 months and 12 months after enrollment. Data collection will be integrated into routine primary care. We will also ask all participants to sign a release giving researchers access to their medical charts so that we can pull data from the medical record every 3 months. In addition to the surveys, we will ask patients to complete STI tests and oral, rapid HIV tests. Participants will be able to complete HIV and STI testing at baseline, which will be done as part of routine lab work. Because data collection is integrated with routine patient care, participants will not be paid to be part of the study.**

**The study will be the first U.S. study to evaluate the effects of medical gender affirmation intervention on sexual health outcomes (such as HIV prevention/HIV care, STIs) and other health outcomes (like mental health, substance use, quality of life, and chronic disease management) across 12 months of follow-up in a large diverse TG patient cohort. Findings will directly improve the quality of the evidence available to help patients, providers, and relevant stakeholders make informed health decisions and improve outcomes for TG people.**

**3. STUDY PARTICIPATION: Now, let’s talk about study participation.**

- *What are your initial thoughts after hearing more about the study?*
  - *What else would you like researchers to learn about the health of the patients in this study?*
- *We will be integrating this study into routine patient care. What do you think of this approach?*
  - *What are the strengths and weaknesses to this approach?*
  - *Participants will not be paid (incentive) as part of this study. What are your thoughts about this approach?*
- *How do you feel about researchers requesting access to patients medical records every 3 months in order to collect data?*
  - *What are your thoughts on researchers gathering data on sensitive health information such as mental health diagnoses, HIV/STI testing, abortion history, etc.?*
- *The researchers are considering whether or not to ask participants to collect biological specimens (such as blood or plasma) to store for future research, including potential genetic research. How do you feel about this?*

**4. STUDY MATERIALS AND COMMUNICATIONS. We want to ensure the study name and materials are appealing and reflect the community.**

**NOTE TO FACILITATOR**: Display any draft names and logos to obtain feedback.

- ***What types of study materials would you like to see?***
  - ***Thoughts about the study name?***
  - ***Ideas for study recruitment materials?***
  - ***Study images?***
  - ***Logo/symbol?***
  - ***Branding?***
  - ***What would you like to see communicated to potential study participants?***
  - ***What would you like to see communicated to transgender communities more broadly about the study?***

**3. RECRUITMENT: OK, now let’s think about study recruitment. Study recruitment refers to activities to find new people to join and participate in a study. There are many ways to recruit participants in any research study.**

- *What recruitment strategies do you think might be successful to recruit transgender patients to the study?*
  - *What do you see as some barriers (things that make it more difficult) to recruiting transgender patients (using these approaches)?*
  - *What do you see as some facilitators (things that will make it easier) to recruit transgender patients using these approaches)?*

**6. RETENTION: OK, now let’s brainstorm about study retention. Study retention refers to retaining (or engaging) participants in a research study over time. For example, we would like to engage transgender patients in our study for a period of 12 months.**

- *What are some strategies that might be effective to help transgender patients stay engaged in a research study for 12-months?*
  - *What are some barriers (things that might make it more difficult) to keeping transgender patients engaged in a study for 12-months?*
  - *What are some facilitators (things that might make it easier) for transgender patients to stay engaged in a study for 12-months study?*
- *Any other suggestions for retention strategies?*

**7. ENDING.**

Is there anything else that wasn’t already discussed that you’d like to share with us about these topics?

OK, this concludes the discussion. We appreciate you participating and sharing your valuable perspectives.

**[TURN OFF DIGITAL RECORDER]**

**NOTES:**
